# Parents' experiences in accessing services for their autistic children in the United Kingdom: A meta‐synthesis

**DOI:** 10.1111/bjc.70033

**Published:** 2026-01-07

**Authors:** John Kerr, Hannah Nicholson, Rhiannon Richards, Ciorsdan Anderson

**Affiliations:** ^1^ Department of Experimental Psychology Oxford Institute of Clinical Psychology Training and Research, University of Oxford Oxford UK; ^2^ Oxford Health NHS Foundation Trust Oxford UK; ^3^ Berkshire Healthcare NHS Foundation Trust Bracknell UK

**Keywords:** ASD, autism, parents, qualitative, review

## Abstract

**Background:**

Parents of autistic children support their children through additional challenges, often experiencing adversity as a result. Such parents report high support needs, yet service provision is often limited. Services often support children through providing various psychological interventions to parents. Quantitative evidence for such interventions is mixed and qualitative evidence is sparse. This review therefore aimed to synthesise the perspectives of UK parents regarding interventions for their autistic child.

**Method:**

The databases Scopus, Embase, Medline, PubMed, PsycInfo, CINAHL, Web of Science and ASSIA were searched in February 2025. Inclusion criteria constituted qualitative articles published in English from 2004 onwards exploring UK parents’ perspectives of interventions aimed at supporting autistic children. Articles were evaluated using Standard Quality Assessment Criteria. Thematic meta‐synthesis was conducted.

**Results:**

Fourteen papers were identified: eight high‐quality, one medium‐quality, and four low‐quality. Interventions were psychoeducational behavioural, communication‐based, sensory‐related or mental‐health based in nature. Themes included change, relationship with help, parents' need to process and solidarity.

**Conclusions:**

Facilitators of positive change included learning, empowerment, structure and rigour, while barriers included delivery issues and unhelpful information. Parents reported finding solidarity amongst similar parents helpful. Reflective space was deemed useful in facilitating new understanding of autistic lives. Methodological quality varied, with more reflexive and theoretically grounded research encouraged. Future research should also consider implementing embedding processes into qualitative designs.

**Practitioner points**

1. *Facilitating Positive Change*
  1.1 Offer substantive and novel learning opportunities.
  1.2 Design appropriately structured interventions with rigorous elements.
  1.3 Include opportunities for empowerment throughout interventions.
  1.4 Provide clarity regarding intervention processes.
  1.5 Develop evidence‐based inclusion and exclusion criteria and communicate these to parents in advance.
2. *Supporting Parent–Staff Relationships*
  2.1 Parents consistently voiced appreciation for such interventions—continue to offer them.
  2.2 Parents voiced appreciation for empathy and acknowledgement of the difficulties they experience navigating complex support systems by staff.
  2.3 Consider means of supporting parents following their child's ASD diagnosis and parents waiting for interventions.
  2.4 Should autistic children not meet inclusion criteria, cross‐service signposting should be clearly communicated both to parents and between services.
  2.5 Adapt intervention environments so that they are neurodiverse‐friendly.
  2.6 Co‐produce interventions for parents of autistic children.
3. *Promoting Resolution and Solidarity*
  3.1 Include reflective components in interventions that facilitate parental resolution of their child's autism.
  3.2 Facilitate opportunities for connection, mutual validation and practical support between parents.

## INTRODUCTION

Parents of autistic children love and cherish their children just as any parent (Aylaz et al., 2012). However, research has long depicted the additional challenges in raising their children (Bonis, 2016). Such parents are often compelled by necessity to become passionate advocates for the needs of both their autistic child and their families, despite resultant impact on their personal wellbeing, familial relationships and careers (Lee et al., 2021; Smith‐Young et al., 2022; Stoner & Stoner, 2016). Such effort can lead to increased risk of developing clinically significant mental health problems, including depression, anxiety and persistently elevated stress (Cachia et al., 2016; Falk et al., 2014; Machado Junior et al., 2016).

Literature regarding the difficulties children with autism and their parents face in accessing appropriate service support is widespread (Corcoran et al., 2015). Racial and socioeconomic disparities in service access exist for such families, relating to care access, referral frequency and proportion of unmet need (Smith et al., 2020). Fragmented service provision, inadequate service communication and long waitlist periods are commonly reported by parents (Khanlou et al., 2017). Post‐diagnostic support provision has been identified within United Kingdom (UK) services as a systemic limitation (Crane et al., 2018).

Brice et al. (2021) note that the UK's National Health Service (NHS), as a freely accessible provider, differs from service provision for autistic people in other countries, and that barriers to accessing such services duly differ. As Smółka (2022) notes, whilst the NHS is largely publicly funded, with the state and statutory agencies strongly involved in service planning and provision, other countries' healthcare systems are designed differently; for example, health care in the United States is largely market‐based. Therefore, service design, spending, cultural context and staffing contribute to varying international efficacy in supporting service users (Anandaciva, 2023). The UK Government has recognised that many autistic children remain on waiting lists for longer than the maximum of 13 weeks outlined in clinical guidance, attributing diagnostic and treatment delays to factors such as increased referral rates, local system issues caused by the COVID‐19 pandemic and inefficient diagnostic pathways within services (Department of Health and Social Care & Education, 2021).

Furthermore, dramatic increases in prevalence and incidence of paediatric ASD diagnoses have been observed in the past 20 years (NHS England, 2023; Taylor et al., 2013). In this time period, several legislative acts and policy frameworks have been introduced that have drastically reformed recognition and intervention for autistic children. These include the UK Government's 2009 Autism Act (UK Parliament, 2009), which focused on improving service access, promoting independent living and developing clear support pathways, clinical guidance on the recognition and diagnosis of autism in young people (NICE, 2013), and the introduction of a national strategy for autistic children, young people and adults that specifically aims to improve treatment pathways in Child and Adolescent Mental Health Services (CAMHS) (Department of Health and Social Care & Education, 2021).

The UK's National Institute for Health and Care Excellence (NICE, 2013) recommends various forms of therapeutic support for autistic children and their families. These include behavioural approaches such as Applied Behaviour Analysis (ABA) and Early Intensive Behavioural Interventions (EIBI), communication therapies including Social Communication Therapy and Paediatric Autism Communication Therapy, sensory support interventions, such as Sensory Integration Therapy, and a variety of psychologically therapeutic interventions including psychoeducational groups and various forms of psychological therapy for children and families (Cooper et al., 2018; Denne et al., 2017; Green et al., 2018; Pickles et al., 2016; Randell et al., 2019; Roughan et al., 2019).

Such interventions achieve optimal results when targeted and intensive (Nevill et al., 2018). However, logistical challenges on the part of parents and services, ranging from time limitations to long waitlists, create barriers to such intervention on the part of services (Jimenez et al., 2012). While parents are therefore well‐placed mediators of such interventions, review and meta‐analysis have identified that the efficacy of such interventions is inconclusive; disparities between clinically relevant improvements in parent‐rated adaptive functioning contrast with a lack of similar clinician‐rated improvements (Conrad et al., 2021). The components involved in successful interventions remain unclear (Trembath et al., 2019)—and to date, no review has collected UK parents' perspectives or opinions on what components were useful or unhelpful.

Similarly, the evidence base on direct treatment of psychological disorders for autistic children showed significantly superior parent‐ and clinician‐reported outcomes post treatment compared to waitlist and treatment‐as‐usual controls; however, there was no significant difference in post‐treatment self‐reported outcomes (Kreslins et al., 2015). Similar results were found in a meta‐analysis by Wang et al. (2021) of Cognitive Behavioural Therapy (CBT) for a range of social–emotional problems, including depression. Recent meta‐analysis of CBT and social‐skills therapies for ASD populations illustrated reductions in child anxiety in the former, and both anxiety and depression in the latter (Wichers et al., 2023). While exposure (Guzick et al., 2022) and emotional regulation skills (Helland et al., 2023) represent potential mechanisms of change, Luo and McAloon (2021) note that research delineating components of effective psychological therapy for autistic children is limited.

To date, much of the qualitative evidence base regarding parents' interactions with UK services for autistic children has focused on either experience of the ASD diagnostic process (Legg & Tickle, 2019) or service access (Babalola et al., 2024). Similar trends are apparent globally (Boshoff et al., 2021; Naicker et al., 2023; Smith‐Young et al., 2025), while parents' experiences of ASD assessment processes (Howes et al., 2021), stigma (Mitter et al., 2019) and providing care (Corcoran et al., 2015; DePape & Lindsay, 2014) have also been explored further afield.

However, while the quality of support provided by relevant UK services has long been criticised (Read & Schofield, 2010; Tissot, 2011), no review to date has summarised parents' perspectives on interventions provided to their autistic children. This review therefore aims to, using a meta‐synthetic approach, collate the perspectives of parents raising autistic children regarding their experiences of interventions in the United Kingdom. It shall examine qualitative studies based in the United Kingdom. It is intended that this review will provide a helpful resource for clinical psychology professionals providing therapeutic interventions for autistic children and that future interventions may be better informed by the lived experience of parents of such children.

## METHOD

Given markedly increased rates of diagnosis and assessment in the 20 years preceding this study (NHS England, 2023), this review examines studies published from 2004 onwards to ensure that themes elucidated reflect the modern clinical context.

### Reflexivity

Prior to establishing search criteria, a bracketing interview between the main researcher (J.K.) and their research supervisor (C.A.) was held. This interview explored potential biases, assumptions and beliefs held by both researchers. J.K. contextualised himself within this research as a sibling of an autistic young person with experience navigating support services, as an immigrant, and as a psychological practitioner with therapeutic relationships with autistic youths and published research in the subject area (Kerr et al., 2023).

### Inclusion criteria

Articles for inclusion comprised studies with qualitative data depicting the perspectives, experiences, or perceptions of UK‐based parents of autistic children regarding interventions aimed at supporting the young person psychologically, whether parent‐, child‐ or family‐focused. Psychological support was deemed any support related to mental health, communication, behaviour or sensory integration. For the purposes of this study, autism was characterised in line with the Diagnostic and Statistical Manual of Mental Disorders, Fifth Edition; this included diagnoses of Autism Spectrum Condition, Asperger's Syndrome, autistic disorder and pervasive developmental disorder not otherwise specified. Studies using the International Classification of Diseases, 11th revision or parental confirmation of autism diagnosis were also deemed acceptable for inclusion.

### Exclusion criteria

Articles excluded were those not translated to English, those based outside the United Kingdom, those with quantitative analysis only, those that focused on interventions received by autistic offspring in adulthood, those that mentioned neurodiversity or developmental disorder but did not specify autism, those with parents of young people solely with learning disabilities and those solely featuring the perspectives of parents with regard to siblings of autistic children.

### Search strategy and screening process

This review was prospectively registered on Prospero by the main researcher. Six databases were searched from 2004 up to 2025 for articles involving parents' perspectives on psychological interventions for their children with autism. Databases searched included Scopus, PsychInfo, PubMed, EMBASE, Web of Science, Applied Social Sciences and CINAHL (see Appendix A for search terms). J.K. also manually searched Google Scholar and relevant reference lists for additional potentially eligible articles. Following the removal of article duplicates, titles and abstracts were screened by J.K.

**FIGURE 1:**
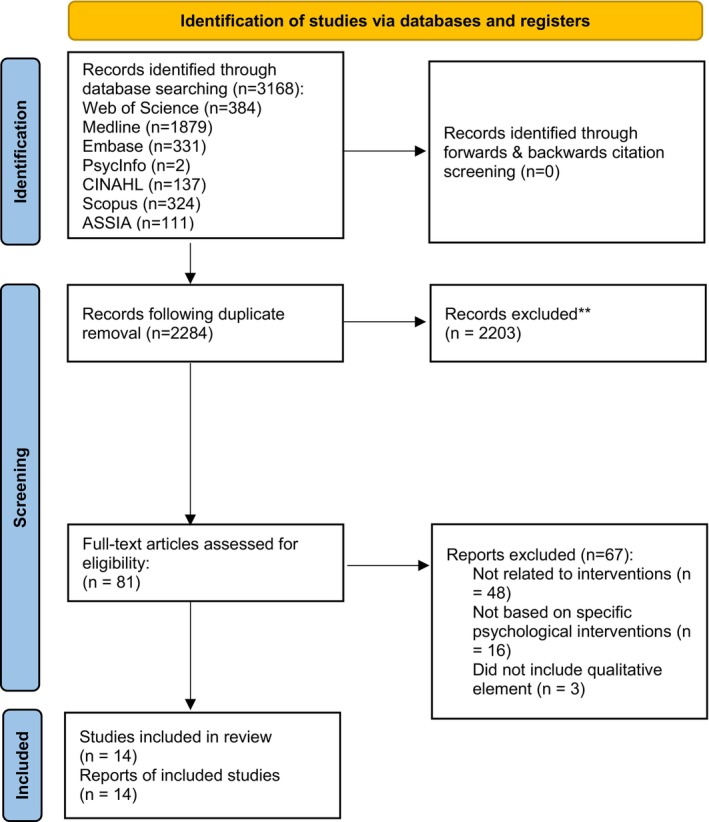
Systematic review search and screening process based on the PRISMA 2020 statement.

A Preferred Reporting Items for Systematic Reviews and Meta‐Analysis (PRISMA) flowchart depicts the review process (see Figure 1). The combined electronic database searches retrieved 3168 results. Following removal of duplicates, 2284 papers remained. All titles and abstracts were reviewed by JK (JK). Of these, 20.77% (658 papers) were also screened by HN (Cohen's *k* = 1). If papers did not meet inclusion criteria, they were excluded from further review. This excluded 2203 papers.

The full text of 81 papers were reviewed. From full‐text review, a further 68 were excluded. CA (a clinical psychologist experienced in working with parents of autistic children) reviewed the final 14 papers to verify eligibility.

### Data extraction

Relevant information extracted included title, author, publication year, origin country, type and length of intervention, sample size, methods of data collection and analysis, and themes.

### Quality assessment

Studies' methodological quality was established using the qualitative component of the Standard Quality Assessment Criteria for Evaluating Primary Research Papers from a Variety of Fields (Kmet et al., 2004). This tool was chosen for its applicability to a range of study designs and analytic approaches. Papers were individually evaluated against the tool's checklist to analyse risk of bias and quality scores were attributed accordingly. J.K. & R.R. independently conducted quality appraisals for all included studies, with discrepancies in quality ratings resolved through discussion. Fair agreement between the two reviewers, *k* = .34, 95% CI .059 to .619, *p* < .001, was initially found. A summary of study quality is included in Table 1.

**TABLE 1:** Characteristics of studies included.^a^

Author(s)	Year	Published	Intervention type	Intervention length	Sample size (*n*)	Study type	Data collection methods	Analytical framework	Main themes	Study quality
Trudgeon & Carr	2007	Yes	Early Intensive Behavioural Interventions	Varied	16	Qualitative	Semi‐structured interviews	Grounded Theory	Search for Treatment; Establishing ABA Programme; Insights; Impacts; Perceived Support Needs	13
Grindle, Kovshoff, Hastings et al	2009	Yes	Home‐based ABA Programme	2 years	53	Qualitative	Semi‐structured interviews	Content Analysis	Practical benefits; practical difficulties; impact of EIBI on family relationships; emotional impact of EIBI; overall evaluation of EIBI	14
Cuttress & Muncer	2014	Yes	Psychoeducational Parent Group	10 weeks	120	Qualitative	Questionnaire	Content Analysis	Learning about autism, learning about communication, learning about behaviour, messages for others	16
McAleese, Lavery & Dyer	2014	Yes	Psychoeducational Parent Group	3 sessions	83	Mixed‐methods	Questionnaire	Thematic Analysis	General positive talk; felt security and support; timeframes; workshop content and application	14
Peckett, MacCallum & Knibbs	2016	Yes	Social Communication Therapy	6 sessions	15	Qualitative	Semi‐structured interviews	Interpretive Phenomenological Analysis (IPA)	Communication; new perspectives; more appreciative/interactive sibling relationships; ASC and Lego Therapy fit; Child specific Developments/ barriers (time, ambivalence)	15
Hodgson, Grahame, Garland et al	2018	Yes	Psychoeducational Parent Group	8 weeks	14	Qualitative	Focus Groups	Framework Analysis	Experiences of participating in a RCT, opinions about the intervention and the impact of the intervention on the participants, their children and the family.	13
Jackson, Keville & Ludlow	2020	Yes	Child and Adolescent Mental Health Services	Varied	7	Qualitative	Semi‐structured interviews	IPA	Psychological impact on caregiver, negative access experience, relationship breakdown with professionals	18
Leadbitter, Macdonald, Taylor et al	2020	Yes	Paediatric Autism Communication Therapy	18 sessions	18	Qualitative	Semi‐structured interviews	Thematic Analysis	Backdrop to therapy, therapeutic process, practical challenges, therapeutic outcomes	19
Palmer, Cáceres, Tarver et al	2020	Yes	Psychoeducational Parent Group	8 sessions	17	Mixed‐methods	Semi‐structured interviews	Thematic Analysis	Increased knowledge; improvements in stress and wellbeing; supportive environments; changes in children; relevance of content; additional content; ideal group size; lack of time	15
Rodgers, Goodwin, Garland et al	2023	Yes	Parent‐Supported Group Mental Health Intervention	8 weeks	50	Mixed‐methods	Semi‐structured interviews	Thematic Analysis	Acceptability; impact	12
Ashworth, Bray, Hanlon et al	2025	Yes	Child and Adolescent Mental Health Services	Varied	300	Mixed‐methods	Survey	Content Analysis	Experiencing mental health difficulties; seeking mental health support; first CAMHS appointment; provision of support	15
Leadbitter, Harrison, Langhorne et al	2024	Yes	Psychoeducational Parent Group	5 weeks	24	Mixed‐methods	Semi‐structured interviews & Focus Group	Thematic Analysis	Broad acceptability, accessibility issues, perceived benefits	15
Pettitt	2024	No	Family‐Based Treatment for Anorexia Nervosa	Varied	13	Qualitative	Semi‐structured interviews	Abductive Framework Analysis	Interplay between ASC and current treatment methods; Relational aspects of treatment; Issues encountered during treatment and recovery	18
Randell, McNamara, Busse et al	2024	Yes	Sensory Integration Therapy	26 weeks	30	Mixed‐methods	Semi‐structured interviews	Framework Approach	Overall experience; trial info; completing measures/assessments	13

^a^ One study was excluded as it did not meet minimum quality thresholds.

### Data synthesis

Fourteen papers were reviewed following a meta‐synthetical approach, as described by Lachal et al. (2017). Meta‐syntheses facilitate deep and broad explorations of phenomena by converging disparate findings on the subject matter (Hoon, 2013), supporting this study's aim to establish parents' overarching lived experiences of their autistic children's receipt of psychological intervention.

Each study was first actively read before line‐by‐line coding was conducted on the full qualitative segments of each included study. These codes were then grouped into hierarchical structures before analytical themes were generated. J.K. discussed their perceptual biases and opinions in regular supervision with C.A.

## RESULTS

### Study information

Results included 14 papers published between 2004 and 2024. Methods of data collection included semi‐structured interviews (8), questionnaire items (3) and focus groups (3). Sample sizes varied from 8 to 120. Type of interventions included psychoeducation (6), behavioural (2), communication (3), mental health (1) and sensory integration (1). They ranged from 3 to 26 weeks in length. Participants were predominantly recruited from the service in which research was being conducted.

None of the studies included in this review achieved maximum scores using the Standard Quality Assessment Criteria for Evaluating Primary Research Papers from a Variety of Fields (Kmet et al., 2004). Eight studies were considered of high quality, one medium quality and four low quality. One study—Roberts and Pickering (2010)—was excluded as it did not meet the minimum threshold of 55% suggested by Kmet et al. (2004).

### Meta‐synthesis

**FIGURE 2:**
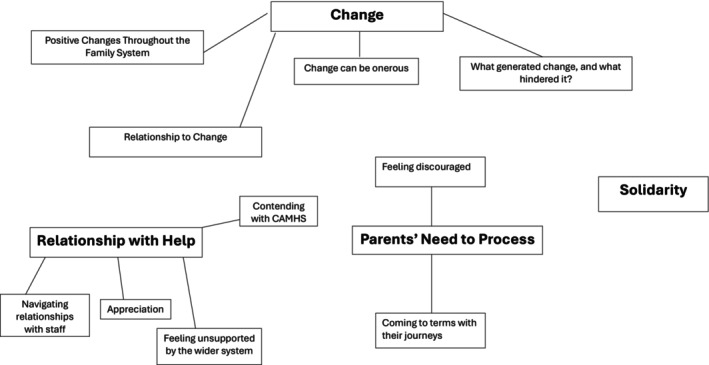
Thematic map.

Four themes were developed through meta‐synthesis: change, relationship with help, parents' need to process and solidarity (see Figure 2).

### Theme 1: Change

#### Positive changes throughout the family system

Nearly all of the papers included in this review explored the variety of positive changes invoked through participation in the interventions offered. Many of these were *positive changes for the autistic child*, including improved capacity to communicate with others, greater social integration, better awareness of their own behaviour and enhanced emotional regulation skills. *Positive changes for parents* were also apparent throughout; they described increased optimism regarding their child's prospects, more confidence in supporting their child appropriately, having improved wellbeing and social connectivity, and better capacity to manage difficult emotions regarding parenthood. *Positive changes in family relationships* were relayed by many parents. This could include relationships between siblings who were often typically developing; parents reported their children were ‘squabbling less’, and the autistic child being seen ‘in a more positive way’ (Peckett et al., 2016). Some parents described positive developments in their marriages; drawn ‘closer together as a couple’ or by making partners ‘more aware of the way you speak and how you convey information’ (Peckett et al., 2016; Trudgeon & Carr, 2007). An improved capacity to partake in endeavours as a familial unit was discussed in several papers (Peckett et al., 2016; Trudgeon & Carr, 2007); for example, improvements brought about by interventions facilitated relational activities ranging from play (‘We tend to avoid confrontation and we tend to split them up, and I think doing this now, I realise that it's not always necessary’.), to social outings (‘Now we are able to go out and do the things as a family that were so restricted in before’.), to family holidays (‘I think it's the autism that affects plans for a holiday. In the past he used to get so upset with change but now it's not such an issue’.). Closer dyadic relationships between parents and autistic children were explored in many of the studies included; as one parent described it, ‘I have always loved my son… but I fell in love with him after this’ (Leadbitter Macdonald et al., 2020). Many studies attributed this greater closeness to a clearer understanding of their child; whether better recognising their emotions and reacting appropriately (‘I now realise I used to think X was being naughty and tell him off … if he gets upset/anxious I react differently now’.), greater capacity to take their child's perspective (‘I put my eyes behind [child's name]'s eyes and I just imagine for a minute that I am in [child's name]'s little head and try and look at it from his angle … and you probably react totally different to the way you would initially react, because you have looked at it from his point of view’.) or having more constructive attitudes towards their child's difficulties (‘He's always going to have these challenges, it's more how you adapt around him, how you deal with that, rather than making it go away’.) (Cutress & Muncer, 2014; Hodgson et al., 2018; Leadbitter Macdonald et al., 2020).

#### What generated change: And what hindered it?

Most papers explored the components of interventions that acted as barriers and facilitators to change for the autistic child and their family. Various *facilitators of change* were discussed across the studies. Learning new things was commonly described as conducive to improvements; whether that was how to become more aware of one's own behaviour, using techniques such as Icebergs or social stories, developing greater understanding of non‐verbal communication, or the importance of routine to their autistic child. Structure was another component parents viewed as beneficial in various elements of the interventions they participated in; this could entail receiving sufficient information regarding the intervention prior to beginning, knowing clear rules regarding homework, or being given clear explanations as to why a task may be beneficial. Relatedly, rigour was received positively across various studies; while the intensity of intervention workloads could be high, they were ultimately viewed positively for that very reason, as one parent explained: ‘It was like you was being analysed, everything you'd just done, was like she was like ripping it all to bits and putting it in little [chunks], and I didn't like that, but it was good in the end’ (Leadbitter Macdonald et al., 2020). Parents reflected that their own high level of commitment was conducive to the change generated by interventions: ‘I think if we hadn't have done that, I think the homework and stuff, that you wouldn't have got very much out of the course’ (Hodgson et al., 2018). *Empowerment* was also highlighted as an ingredient of success; knowing that you are taking a constructive approach can instil confidence, as a parent conducting EIBI explained: ‘I think parents who are involved in this type of programme are more empowered… and I think it takes a lot of the anxiety away’ (Trudgeon & Carr, 2007). Conversely, various *delivery issues* were also identified. Factors such as therapist unavailability, or lack of clarity from intervention providers, caused issues for parents; studies featured setbacks such as participants not being told what group their child was in, not knowing what the role of involved professionals were, or professionals not attending all relevant meetings. While learning was viewed as important, parents were sometimes frustrated by low quality of learning material, such as the parent reporting that the ‘stuff we got was a bit late and it just, it just wasn't that good… I found it a little bit airy fairy…’ (Pettitt, 2024). Additionally, the issue of *biased selection* was raised by one parent, who mused ‘They can pick and choose whether they want children in their programme… maybe it's easier to choose easier children’ (Pettitt, 2024).

#### Change can be onerous

Across each study, differing perspectives on the demands placed on parents by intervention participation were explored. *Intensive workloads* were sometimes reported by parents; these ranged from completing long questionnaires to videotaping their child's behaviours to managing therapy teams during home‐based treatments. As one parent summarised, ‘…as the weeks go on you need more and more things—copying the recording sheets, preparing for workshops, getting pictures together of things to teach the child, it can all take a lot of time’ (Trudgeon & Carr, 2007). *Logistical burdens*, such as time commitment and the travel required to access treatment, were also commonplace across studies; these challenges were compounded by the additional needs of their children. One parent explained: ‘I just felt the sessions were a bit long… they could be an hour and a half… it's just [child's name] is very on the go, so it's quite difficult’ while another noted, ‘He didn't like the small [therapy] room… he started, like, screaming and not wanting to go in…’ (Leadbitter Macdonald et al., 2020). Other parents, leading busy lives, struggled to engage with interventions due to time restraints—as one mother described, ‘I was trying to work as well and I don't have much time for it’ (Palmer et al., 2020). Some parents reported *implementation challenges* between sessions; these included children's outbursts disrupting parents' strategies, parents not having sufficient time to take notes required for future sessions, or finding homework emotionally difficult to complete as it involved focussing on aspects of their child, they found negative. Furthermore, parents did not always appreciate the rigidity of treatment plans; as one mother remarked, ‘it's not one thing's gonna fit all. It's not gonna fit every single family’ (Pettitt, 2024).

#### Relationship to change

How participating parents viewed change varied across studies. Many parents wished that *change could have happened sooner*; as one parent noted, ‘when we heard the news, we were left to deal with it… this would have helped greatly’ (McAleese et al., 2014). The entrenchment of unhelpful approaches to supporting their autistic child, the solitude parents felt in supporting their autistic child through their struggles, and the anxiety felt by their children without appropriate support were all offered as examples of challenges avoidable through quicker intervention. Throughout interventions, some parents *wanted change quicker*, frustrated when presenting problems were not rapidly resolved. One parent reflected they had believed ‘they're going to give me some miracle potion or something’ (Leadbitter Macdonald et al., 2020). Some were *unsure whether change resulted from intervention*, noting that while their child was in a better place, it was unclear whether that development could be attributed to the intervention offered; for instance, one parent noted ‘he started to talk, so that was a big thing—but that could have been his age’, while another engaging in Lego Therapy mused that positive change was ‘probably a combination of him being generally happier and… maybe the Lego’ (Leadbitter Macdonald et al., 2020; Peckett et al., 2016). Reflections on *maintaining change* arose in several papers; while some reported that change had been sustained following interventions' conclusion, others reported struggling to continue implementing strategies practiced during intervention. As one described, ‘it's just because you let things [go], whereas you were going there you had to keep doing like your little bits of things that you were doing, you sort of slide out of that a little bit now’ (Leadbitter Macdonald et al., 2020). Others found it difficult to maintain changes given the difficulties their children faced at the time of intervention; one parent reflected that ‘given the circumstances, if the circumstances had been different, and [name] hadn't been going through his issues, it probably would have had a bigger impact’ (Peckett et al., 2016).

### Theme 2: Relationship with help

#### Navigating relationships with staff

Many papers explored the relationship between the parents’ receiving interventions and the staff delivering them. Some parents described resolute emotional support from therapists or facilitators. As one described, ‘the speech therapist, she was lovely, she was absolutely fantastic… it was difficult … and she saw how difficult it was’ (Leadbitter Macdonald et al., 2020). In several studies, parents celebrated affirmative, nurturing and supportive environments cultivated by staff, which for some marked a change from previous experiences of supporting their child in solitude: ‘Initially it felt like a huge support as previously I had been left at home to manage him alone and there was so little you could do with him’ (Trudgeon & Carr, 2007). Some parents described positive relationships with staff, but struggled with their intensive nature in the context of the interventions. As one summarised, ‘We trust them implicitly, but at the same time, you don't want your best friend with you all the time do you?’ (Trudgeon & Carr, 2007).

#### Appreciation

Across many of the studies included, parents expressed their gratitude for the work invested into the interventions offered. Having therapists with specialist insight was described by one parent as ‘a gift’ (Rodgers et al., 2019), while another offered effusive praise for staff members offering guidance. Nearly half of the studies included offered parents' explicit endorsement of the interventions they participated in.

#### Feeling unsupported by the wider system

Conversely, various parents described experiencing a lack of support from at least one component of the care systems designed to support their autistic child. Parents described experiencing a desperation from support throughout their autistic child's upbringing—as one mother explained, ‘I just feel like I've been banging my head against a wall actually I'm going around in circles I keep being sent back to where we started, and they say, no we can't help, so on to the next bit who then send us back. No one listens’ (Jackson et al., 2020). Some noted a lack of support from their child's school, with one parent outlining that ‘schools need to be much more aware, flexible and understanding of needs’ (Ashworth et al., 2025). For others, local authorities denied them important funding. Some found support systems rigid; as one parent described, ‘If you wanted to do anything that was outside of this little box it was like “Oh no, we can't let you do that”’ (Grindle et al., 2009). Others felt desolate in their isolation—one parent recalled thinking ‘I thought I can't manage this on my own anymore’, while another lamented that professionals seemed to not understand the extent of their autistic child's difficulties: ‘you have had a child who has been hysterically sobbing and banging their head against the wall or harming themselves or wanting to throw themselves out of the window. Do you know what that's like…I don't think they do’ (Jackson et al., 2020; Pettitt, 2024). The sense of needing to prove that support was required proved frustrating for parents across many of the studies; as one father summarised, ‘You always have to justify yourself’ (Trudgeon & Carr, 2007).

### Contending with CAMHS

Several studies depicted parents' struggles to access adequate mental health treatment for their autistic child from CAMHS. In these studies, parents described long waits for their child's treatment. These waits were usually arduous as parents watched their child's mental health deteriorate, as one mother described: ‘we don't know how to support her—we were just looking at the letter on the notice board for months’ (Jackson et al., 2020). Parents' sense of impending crisis was prevalent in such studies; one reflected that ‘you just sit on lists until things get so bad they have to help, or they're too late and another young person loses their life’ while another recalled thinking ‘I need the help now …what do I do now? How do I get through these next few months?’ (Ashworth et al., 2025; Jackson et al., 2020). Alongside such waits, parents reported grappling with high diagnostic thresholds that sometimes excluded autistic children entirely. One parent reported a service wouldn't see their child ‘because she's autistic so we had 6 months of them arguing between themselves’, while another was told that a CAMHS service ‘don't deal with autistic children’ (Ashworth et al., 2025; Jackson et al., 2020). Several reported that treatment was offered only at crisis point; parents reported being ‘told she doesn't meet the criteria as she isn't in crisis or harming herself’, another reflected that ‘it's not until you become critical that you actually get some help’ and a third said they were ‘only listened to when the self‐harm and risk of suicide escalated’ (Ashworth et al., 2025; Pettitt, 2024). Parents' perceptions of their child's mental health treatment varied. Some described positive experiences; one mother reflected that her child ‘made a real brilliant recovery, which is maybe testament to family based therapy’, while another described CAMHS staff as ‘the first professionals who actually identified the real problem and solution’ (Ashworth et al., 2025; Pettitt, 2024). Others, however, described less helpful encounters; for instance, one parent described a clinician as ‘very text book, very wooden… it almost felt like she was going home and reading up on CBT strategies’ (Jackson et al., 2020). Lack of accommodation for autistic children was described in several studies; one parent stated that their ‘daughter was forced into a meltdown when the nurse asked around 50 direct questions on the first appointment despite [them] explaining she wouldn't cope’, while treatment environments were not always hospitable to sensory differences, as summarised by another parent: ‘being in a different room each time and very noisy building. She'd be… distracted with people walking past’ (Ashworth et al., 2025; Pettitt, 2024). These studies included recommendations for service improvement. These included screening for autism in CAMHS: ‘anybody who goes to CAMHS, the minute they hit their system they should be looking at that’, offering support immediately following diagnosis: ‘there is nothing post‐diagnosis which is awful… there should be a separate mental health/autism team who support families once diagnosed!!’, alternative therapeutic approaches: ‘CAMHS causing more harm by offering therapies which are inappropriate e.g. CBT, DBT, PBS—There needs to be … therapies to let them express freely, not made to conform’ and simply validating difficult experiences: ‘Please, just acknowledge there's an issue’ (Ashworth et al., 2025; Jackson et al., 2020; Pettitt, 2024).

### Theme 3: Parents' emotional upheaval

#### Interventions invoke difficult emotions

Various parents reported difficult feelings that arose throughout interventions. Some reported waning motivation as the intervention progressed as a result of emotional turmoil; one explained ‘My feelings of motivation fluctuate all the time. Sometimes I get very depressed about the whole thing’ (Grindle et al., 2009). For those who did not find the success they were hoping for their child, disappointment was difficult to process; as one shared, ‘we were hoping his speech would have improved with the intervention, but it didn't…. it just hasn't really happened’ (Leadbitter Macdonald et al., 2020). In another study, guilt was rife as parents reflected on their children's distress, worrying that they had allowed it to escalate. These parents shared sentiments such as ‘I didn't know if I'd done something to make it worse’, ‘I feel I really missed the boat’ and, in reflecting on how they approached support from services, ‘I think I was naïve and just trusted I would be told about all these services that were on offer… I should have pushed not just accepted’ (Jackson et al., 2020).

#### Coming to terms with their journeys

Various parents described the impact raising autistic children had on their personal lives, and their personal processes of accepting the difficulties accompanying their differences. Some missed opportunities to further themselves professionally: ‘It's had more of an impact on my career… I really wanted to do a Master's and stuff, but the reality of trying to do that and cope with (name)…’ (Trudgeon & Carr, 2007). Some parents withdrew socially: ‘We used to stop going places because we couldn't take (child's name)’ (Trudgeon & Carr, 2007). Some witnessed the deterioration of their child's mental health, and experienced their own distress as a result; one mother described being on the ‘verge of a nervous breakdown’ as a result of her child's anxiety (Jackson et al., 2020). For some parents, participating in their intervention invoked acceptance, as their child did not make the progress they had hoped. One described this realization as ‘grieving for the loss of the child we thought we were going to have’ (Leadbitter Macdonald et al., 2020).

### Theme 4: Solidarity

Most papers explored the unique bonds forged in shared journeys by participating parents. As one explained, ‘As a parent of a newly diagnosed child with autism it is ideal to meet others in the same situation’ (Cutress & Muncer, 2014)—parents were able to receive both practically useful advice and emotional validation from others who understood their particular struggles. Remarks such as ‘it was nice to say what you wanted without judgement’ capture the felt sense of relief of parents to share space with others who understood the particular lens they viewed the world through given the unique challenges of raising autistic children (Rodgers et al., 2019). This sense of connectivity was not always felt in day‐to‐day life, as another parent explained: ‘It was nice to talk to other people as well who understood what you were going through, any other parent that, like in the school playground, that you talk to who isn't in same position as yourself don't understand… when you're trying to talk about your children with these people the things are so different between the children’ (Hodgson et al., 2018). Parents across studies explained that these shared spaces facilitated their coming to terms with diagnoses, helped them work out practical solutions and helped them develop good role models for their own unique parenting journeys. Parents identified that their children could benefit from similar shared spaces: ‘We wouldn't have a child with this level of anxiety if they had met peers who were similar to them… They'd feel less lonely and their anxiety would be reduced. They'd be more independent and have improved self‐esteem’ (Ashworth et al., 2025).

## DISCUSSION

This review sought to synthesise the experiences of parents receiving interventions aimed at supporting their autistic children in the United Kingdom from 2004 to 2024. The qualitative results and quality of 14 studies were explored in this review. Quality appraisal established that eight of these studies were of high quality, one medium quality, and four low quality. Results capture components of parents' experiences that may offer useful insights for those designing future interventions for such parents.

### Interpretation and implications

The current review portrayed parents' descriptions of the positive changes they experienced due to the interventions they participated in. Facilitators of positive change included learning, structure, rigour and a sense of empowerment, while identified barriers included delivery issues and unhelpful information. Parents' reporting of positive change corroborates various quantitative reviews and meta‐analyses reporting varying degrees of effectiveness of parent groups for autistic children, particularly improvements for parents, children and parent–child relationships (Deb et al., 2020; Gerow et al., 2018; Li et al., 2024; O'Donovan et al., 2019; Oono et al., 2013). It is perhaps notable that the evidence base substantiating such reported effectiveness has been found lacking, particularly outside the United States; heterogeneity of intervention content, outcome measurement and design, plus low reported study quality, complicate conclusions regarding the effectiveness and generalisability of quantitative papers in this area (Dawson‐Squibb et al., 2020; Deb et al., 2020; O'Donovan et al., 2019). This study offers the novel finding that just under two‐thirds of such studies conducted in the United Kingdom are of high quality. Therefore, our review can support clinicians to design therapeutic interventions for autistic children mindful of the barriers and facilitators to positive change described by UK parents.

Findings regarding the sense of solidarity across the interventions reviewed echo literature extolling the benefits of mutual parental support in this population, including a sense of belonging, benefiting from practical insight, and emotional support (Gillberg et al., 2024; Lee et al., 2024). However, despite these identified benefits and substantive literature consistently describing parents' experiences (Batchelor et al., 2021; Meadan et al., 2010; Moody et al., 2019; Shilling et al., 2013), exploration of how interventions for parents of autistic children can best facilitate such solidarity is scant in the literature. A recent scoping review in the United States demonstrated the utility of peer‐to‐peer support within this population (Lee et al., 2024); our review is novel in highlighting how parental solidarity in group interventions for those raising autistic children could benefit parents.

The emotional processes described by parents in the reflective spaces that interventions offered are similar to the construct of ‘resolution’ as described by Naicker et al. (2023): understanding an autistic child's diagnosis and its implications on parents' schemas of their child. This phenomenon involves adjusting expectations to fit with the reality of raising an autistic child and accepting the consequent emotional journey (Da Paz et al., 2018; Milshtein et al., 2010). Descriptions of frustration and sacrifice across the studies included in this review are echoed in a systematic review of factors related to parental resolution of ASD diagnoses (Naicker et al., 2023). To date, however, no intervention in the literature has explicitly aimed to support parental resolution; one study has explored its association with intervention fidelity (Grogan et al., 2023). To our understanding, this review is the first to conceptually link parents' emotional processing throughout therapeutic interventions for their autistic child and parental resolution of ASD diagnoses. It is hoped that future researchers and clinicians will explore this conceptual link further.

Relationships between parents of autistic children and relevant service providers are well documented in research (Hodgetts et al., 2013; Samsell et al., 2022; Sperry et al., 1999). The opposing paradigms of unsupportive wider systems and appreciation discussed across the 14 studies reviewed are reflected in wider literature; systematic reviews chronicle both the importance of support from diverse services (Wallace‐Watkin et al., 2023) and the positive effects of good interactions with professionals on future provider relationships (Boshoff et al., 2018). Given the notable expertise parents wield through seeking appropriate interventions for their autistic children (Edwards et al., 2018), co‐production could facilitate design of interventions across diverse services by allowing parents direct input into the support such services provide. Early collaborative successes with mental health (Cullingham et al., 2024) and research bodies (Aabe et al., 2019) for parents of autistic children may offer insight into doing so—one study included in our review may be the first of its kind in the United Kingdom (Leadbitter et al., 2024).

### Limitations of review evidence

Methodological quality varied widely, with papers scored between 6–19 out of 20. Notably, only 3 of 14 studies were deemed to offer fully reflexive accounts. Reflexivity in qualitative research typically describes a researcher's process of situating their selves—including their contexts, their opinions and their biases—within the topic they are exploring (Braun & Clarke, 2021). It is particularly important given current challenges in autism research, such as the ideological questions underpinning autism conceptually, the difficulties defining quality of life for autistic people, concerns regarding whether current understandings of autism are fit for purpose, ableism and perceptions of social value pertaining to autism (Botha & Cage, 2022). Such reflective dialogue is important in ensuring practitioners engage with the support of autistic young people meaningfully (Horton et al., 2024).

While all studies at least partially contextualised their research within wider academic literature, only 2 of 14 studies reviewed were deemed as connecting sufficiently to a theoretical framework. As Creswell and Clark (2017) explain, grounding research in theory is pivotal; outlining relevant theory ensures that constructs are defined and measured appropriately and consistently, and facilitates academic exploration of interventions' mechanisms of change.

It is also notable that just over half of selected studies were purely qualitative in design—the rest used mixed‐methods approaches. In itself, this is not an issue—intervention mixed methods frameworks are often used in such research, with qualitative data sought to contextualise an intervention's factors and results relevant to its outcome (Fetters et al., 2013). However, such approaches are advised to implement embedding processes into their study design, whereby qualitative data are used to inform interventions throughout data collection and analysis, for example, establishing appropriate measures, identifying contextual biases and explaining results (Fetters et al., 2013). None of the mixed‐method studies in this review outlined embedding processes used in their research, suggesting missed opportunities to develop more enriched and rigorous interventions.

### Limitations of the review process

The use of the Kmet et al. (2004) quality appraisal tool in this review is susceptible to critiques regarding the use of quality appraisal tools in qualitative research. First, qualitative research spans a wide range of epistemological positions, ranging from relativism to critical realism—critiquing papers from such differing philosophical approaches indiscriminately arguably lacks nuance (Walsh & Downe, 2006). Furthermore, no criteria within the Kmet et al. (2004) tool explore the rigour of data analysis, ethical considerations or saturation—elements all considered by other qualitative quality appraisal tools (Munthe‐Kaas et al., 2019). Finally, quality appraisal tools in general raise questions regarding whether appraisal is based upon the quality of research or the quality of reporting methods; as Braun et al. (2022) note, many studies describe themselves as using Thematic Analysis while not applying sufficient methodological rigour.

## CONCLUSION

Across 14 studies in the period 2004–2025, hundreds of parents participated in qualitative research across the United Kingdom that aimed to capture their experiences of interventions supporting their autistic child. Our review found that these studies, when synthesised, portrayed themes of change, relationship to help, solidarity and reflective space. Study quality varied widely—future research would benefit from stronger theoretical links, more rigorous embedding processes and greater reflexivity displayed by researchers. These findings indicate that learning opportunities, rigour, empowerment and clarity all facilitate positive change. They indicate a need for inclusion and exclusion criteria for the therapeutic interventions discussed and clear communication of these to autistic children and their parents. They highlighted parents' appreciation of such interventions and that parents value the validation and support they provide, while offering suggestions of how such support may be ameliorated (for example, better cross‐service signposting, post‐diagnostic support and opportunities for co‐production). Finally, they highlighted the utility of these interventions as opportunities for parents to find solidarity with one another in the effort to support their children.

## AUTHOR CONTRIBUTIONS

**John Kerr:** Conceptualization; methodology; formal analysis; data curation; writing – review and editing; writing – original draft; visualization; project administration. **Hannah Nicholson:** Validation; data curation; formal analysis. **Rhiannon Richards:** Formal analysis; data curation; validation; methodology. **Ciorsdan Anderson:** Methodology; supervision; writing – review and editing; conceptualization; resources.

## CONFLICT OF INTEREST STATEMENT

The authors have no conflict of interest to declare.

## Data Availability

Data sharing not applicable to this article as no data sets were generated or analysed during the current study.

## APPENDIX A SEARCH TERMS

Search terms used to retrieve articles involved combinations of the following groups of keywords:

1. Parent*, caregiv*, guardian*, mother*, father*, foster*, carer*, famil*;
2. Child*, adolescen*, teen*, young people, youth, boy*, girl*;
3. Focus group*, interview*, qualitative*, exploration, narrat*, experience*, participa*, perspective*, perception*, struggle*;
4. Servic*, support*, provide*, system*, access*, interven*, program*;
5. ASD, Asperger*, ASC, autis*, 47eurodiverse*;
6. United Kingdom, UK, Great Britain, GB.

Boolean operators ‘AND’ and ‘OR’ were used to combine search terms between word sets and connect search terms within word sets, respectively. Truncations ‘*’ were also used to pluralise search terms where possible. Search terms were limited to titles and abstracts in English.
